# PEARLS randomized lifestyle trial in pregnant Hispanic women with overweight/obesity: gestational weight gain and offspring birthweight

**DOI:** 10.2147/DMSO.S179009

**Published:** 2019-02-18

**Authors:** María A Trak-Fellermeier, Maribel Campos, Marytere Meléndez, Jeremy Pomeroy, Cristina Palacios, Juana Rivera-Viñas, Keimari Méndez, Irma Febo, Walter Willett, Mathew W Gillman, Paul W Franks, Kaumudi Joshipura

**Affiliations:** 1 Center for Clinical Research and Health Promotion, School of Dental Medicine, Medical Sciences Campus, University of Puerto Rico, San Juan, PR, USA, kaumudi.joshipura@upr.edu; 2 Clinical Research Center, Marshfield Clinic Research Institute, Marshfield Clinic Health System, Marshfield, WI, USA; 3 Department of Dietetics and Nutrition, Robert Stempel College of Public Health & Social Work, Florida International University, Miami, FL, USA; 4 Department of Obstetrics and Gynecology, School of Medicine, Medical Sciences Campus, University of Puerto Rico, San Juan, PR, USA; 5 Department of Pediatrics, School of Medicine, Medical Sciences Campus, University of Puerto Rico, San Juan, PR, USA; 6 Department of Nutrition, Harvard T.H. Chan School of Public Health, Harvard University, Boston, MA, USA; 7 Division of Chronic Disease Research Across the Life Course, Department of Population Medicine, Harvard Medical School and Harvard Pilgrim Health Care Institute, Boston, MA; 8 Department of Clinical Sciences, Genetic and Molecular Epidemiology Unit, Lund University, Skåne University Hospital, Malmö, Sweden; 9 Department of Epidemiology, Harvard T.H. Chan Public Health School, Harvard University, Boston, MA, USA, kaumudi.joshipura@upr.edu

**Keywords:** gestational weight gain, lifestyle modification, pregnancy, birthweight, neonatal, randomized controlled trial, overweight, obese, intervention

## Abstract

**Background:**

Inappropriate gestational weight gain (GWG) has been associated with adverse perinatal events. High rates of GWG have been reported among Hispanic women. Observational studies indicate that dietary and physical activity interventions during the prenatal period may improve maternal and infant health, but very few randomized trials have been conducted among high-risk overweight/obese Hispanic women. Accordingly, we conducted a lifestyle intervention among high-risk pregnant women and evaluated its impact on achieving appropriate GWG and on improving birthweight.

**Methods:**

Eligible overweight/obese women presenting at the University Hospital in Puerto Rico with a singleton pregnancy before 16 gestational weeks were recruited and randomized to lifestyle intervention (n=15) or control group (n=16). The lifestyle intervention focused on improving physical activity and diet quality and optimizing caloric intake. We evaluated the impact of the lifestyle intervention on achieving appropriate GWG and on infant birthweight. Poisson and linear regression analyses were performed.

**Results:**

The primary intent to treat analysis showed no significant effect on achievement of appropriate GWG/week through 36 weeks in the intervention group (4/15 women) when compared with the control group (3/16 women) (adjusted incidence rate ratio =1.14; 95% CI: 0.20, 6.67). Although not statistically significant, women in the intervention group (6/15) were 1.7 times more likely to achieve appropriate weekly GWG until delivery when compared with controls (4/16 women) (adjusted incidence rate ratio = 1.67; 95% CI: 0.40, 6.94). We observed lower adjusted birthweight-for-length z-scores in the intervention compared with the control group among male newborns with *z*-score difference −1.74 (−3.04, −0.43), but not among females −0.83 (−3.85, 2.19). These analyses were adjusted for age and baseline body mass index.

**Conclusion:**

Although larger studies are required to determine whether women with obesity may benefit from prenatal lifestyle interventions targeting GWG, our results are suggestive of the intervention improving adherence to established Institute of Medicine guidelines.

## Introduction

One in five US women has obesity at the time of conception (23.4%).^1^,^2^ Preconception overweight/obesity increases the risk of gestational diabetes mellitus (GDM) and is associated with long-term weight retention.^3^,^4^ Also, less than one-third of US women meet the Institute of Medicine (IOM) guidelines for gestational weight gain (GWG).^5^ Obesity and hyperglycemia in pregnancy may also influence fetal metabolism and growth through overnutrition.^6^ These putative intrauterine programming events appear to increase fatness at birth and raise the offspring’s risk for future obesity, type 2 diabetes, cardiovascular disease, and premature death.^7^–^9^ In addition, excessive GWG raises the risk of preterm delivery and of maternal cardiometabolic disorders later in life.^10^,^11^

Pregnancy outcomes are also impacted by the interaction of social determinants of maternal health such as race, ethnicity, education, income level, and access to health care,^12^ which in turn influence dietary and physical activity (PA) habits.^13^ Puerto Rico has the highest poverty rate (46%) among US states/territories.^14^ It also ranks first in the prevalence of diabetes (13%) and seventh in hypertension (34.0%).^15^ The five leading causes of infant mortality are also higher in PR than in the US mainland.^16^ In addition, Hispanic women are especially prone to excessive GWG.^17^

Diet and PA-based interventions during pregnancy reduce GWG and lower the odds of cesarean section.^18^ A combined diet and PA intervention during pregnancy yielded modest reductions in GWG (*b*=−1.42 kg, 95% CI −1.89, −0.95) and lower preeclampsia risk, with no reported effects on birth-weight or size; whereas, PA interventions alone appear only to reduce birthweight.^19^ Also, dietary interventions in women with overweight/obesity were shown to reduce GWG (*b*=−2.1 kg, 95% CI −3.46,−0.75 kg), GDM, and preeclampsia risk, but showed no impact on birth outcomes.^20^,^21^ However, few studies have been reported among high-risk obese Hispanic women. Likewise, only limited information is available about the effects of lifestyle interventions during pregnancy on neonatal weight and body composition.

We conducted a randomized clinical trial that evaluated the effects of a diet and PA intervention within an empowerment framework^22^ among Hispanic women, primarily aiming to achieve GWG within the IOM guidelines.^5^ We also evaluated whether the intervention impacted weight-for-length (WFL) *z*-scores at birth.^23^

## Methods

The Pregnancy and EARly Lifestyle improvement Study (PEARLS) is part of the Lifestyle Interventions for Expectant Moms (LIFE-Moms) Consortium, a collaboration among seven clinical centers, a Research Coordinating Unit (RCU), and National Institutes of Health (NIH). The LIFE-Moms Consortium was designed to determine whether various behavioral and lifestyle interventions, reduce excessive GWG and subsequent adverse maternal and neonatal outcomes and obesity in offspring, among pregnant women with overweight/obesity. Specific common measures, procedures and eligibility criteria are consistent across the seven trials,^24^ but each site designed their own study and intervention and defined outcome measures of their interest. We report findings from PEARLS alone here. PEARLS was approved by the University of Puerto Rico Institutional Review Board and by the LIFE-Moms Data and Safety Monitoring Board (DSMB), the latter of which is an independent regulatory group of experts, convened by the National Institute of Diabetes and Digestive and Kidney Disease (NIDDK).^24^ The study was registered in ClinicalTrials.gov [NCT01771133]. Women agreeing to partake in study procedures for mother–infant dyads provided written informed consent.

### Study population and eligibility criteria

Potentially eligible women seeking prenatal care at University Hospital (UH) and attending Women, Infants, and Children (WIC) offices were invited to participate (Table 1).^24^ Women 18 years of age or above who had body mass index (BMI) >25 kg/m^2^, between 8 up and 16 weeks of gestation, and willing to deliver at UH were eligible. History of health conditions that would affect the woman’s capacity to comply with the intervention (i.e., contraindication to aerobic exercise) or have a direct impact on fetal growth (ie, diabetes or multiple pregnancy) were excluded.

### Randomization method and masking

An independent RCU statistician generated an urn randomization scheme.^25^ Study staff other than a designated statistician and intervention staff remained blinded until the trial concluded.

### Intervention

The PEARLS lifestyle intervention was delivered by Registered Dietitians within a health empowerment theoretical framework that promotes individual goal-setting and self-efficacy,^22^,^26^,^27^ through group and individual sessions geared to improve diet and total PA by interrupting sedentary behaviors and promoting frequent movement. The intervention encouraged participants to meet GWG recommendations through monitoring diet, PA, and weight trajectory. The primary focus of the dietary intervention was on total calories.^28^ We provided clear individualized guidelines for food quantity and total calories for distinct pregnancy phases to ensure appropriate GWG. Additional key components of the diet intervention included improving carbohydrate and fat quality, reducing salt and replacing red meat with low-mercury fish, nuts, and beans. Also, the intervention encouraged the use of prenatal multivitamin supplements as prescribed by the participant’s obstetrician.^29^

The primary focus of the PA component was to increase movement and reduce sedentary time. Participants were encouraged to set goals for a daily PA/exercise routine considered safe during pregnancy, according to the American Congress of Obstetrics and Gynecology.^30^ In addition, participants were motivated to increase non-exercise activity thermogenesis by promoting regular movement and encouraging specific behaviors such as standing, walking, parking the car far from one’s destination, self-packing groceries, and taking stairs instead of the elevator.^31^,^32^ For reducing sedentary periods, we recommended minimizing the duration of bouts of sitting or lying during waking hours and interrupting periods of sitting time with 2–5 minutes of activity such as standing or walking.^29^ Additional details of the intervention have been published elsewhere.^29^

### Control group

Women assigned to the control group participated in informative group sessions imparted by study staff, receiving health advice about dental care and child safety.

### Prenatal care

Routine prenatal care continued for all participants at the UH, including advice on maintaining a healthy lifestyle. The majority (94%) of participants were eligible for the Special Supplemental Nutrition Program for WIC and 71% received support (food packages, nutritional and breastfeeding guidance, and anthropometric assessments).^33^

### Assessments

PEARLS was conducted between January 2013 and August 2015 at the UH, the University Pediatric Hospital, and the PRCTRC (Puerto Rico Clinical and Translational Research Consortium). Some visits were completed at the participants’ homes; for these visits, we attempted to complete all assessments as feasible. Trained and certified staff conducted interviews, assessments, and data extraction at <16 weeks (baseline), 24–27 weeks, 6 days, and 35–36 weeks, 6 days of gestational age (GA). Perinatal and delivery data were extracted from medical records. Assessments included height, weight, blood pressure, ultrasound (for dating GA), HbA1c, and triaxial accelerometry. We assessed maternal sociodemographic characteristics, medical and previous pregnancy history, and depression^34^ using questionnaires. We measured height with a wall-mounted stadiometer (Seca^®^ 222, Hamburg, Germany). Maternal body weight was measured (in light clothing; shoes, jewelry, and other objects removed and with an empty bladder) at all study visits, using the same digital scale (BWB-100P, TANITA Corp., IL, USA). In addition, clinical personnel were trained to obtain prenatal weights.

Blood pressure was determined at all visits by a research nurse with an automatic monitor (GE Critikon Dinamap^®^pro 100; GE Healthcare, Chicago, IL, USA), after resting for 5 minutes. Women were clinically screened for GDM between 24 and 27 weeks GA with a 2-hour 75 g oral glucose tolerance test.^35^ Trained interviewers assessed maternal diet using Tucker’s 193 item semi-quantitative Food Frequency questionnaire (FFQ), adapted and validated for PR.^36^ Women estimated the frequency of foods consumption. For baseline, the reference period was the prior year. The time since last FFQ completion constituted the period of reference for each subsequent assessment. Food replicas (Nasco Life/Form^®^, Salida, CA, USA) and standard household serving measures were used as visual aids.

The GT3X+accelerometer (ActiGraph Corp, Pensacola, FL, USA) was used to objectively measure maternal PA. The device records time-varying accelerations (ranging in magnitude from ±6 g) and stores raw acceleration data at a prespecified sampling rate (50 Hz here). Participants wore the accelerometer on the wrist for monthly assessments until delivery. Data collection started with a period of 7 days prior to randomization, followed by 14 days subsequently through delivery. Wear time sufficient to be included in the analyses was defined as at least 4 days per week for at least 10 waking hours per day.

Two examiners took neonatal anthropometric measurements within 1 week after birth, conducting all measurements twice, and a third measure only if the predefined tolerance range was exceeded. The previously established tolerance range was 10 g for weight, 0.5 cm for length, and 0.5 mm for skinfolds. We determined the infant’s nude birthweight with a digital scale (Seca® 354) and used an infantometer (Ellard Instrumentation Ltd., Monroe, Washington, DC, USA) to record recumbent crown-heel length.^37^ Newborn anthropometric measures were performed according to LM standardized protocol within the first 7 days of life.

We measured left side skinfolds, using a Harpenden caliper (Baty International, Burgess Hill, West Sussex, UK). We assessed neonates receiving intensive care if we got medical clearance, and for two infants, extracted weight and length from clinical records (within 7 days after birth). In addition, we measured neck, arm, and thigh circumferences in the women, and head, arm, abdominal and thigh circumferences in the neonates using a Gulick II tape.^37^ Devices were calibrated weekly for maternal assessments and before each neonatal measurement.

### Primary outcome

Average weekly GWG was classified for each woman as “appropriate” or “inappropriate” using the IOM guidelines (for baseline overweight 0.23–0.33 kg/week and for obesity 0.17–0.27 kg/week).^5^ Average weekly GWG was classified from the difference between the weight at the 36-week assessment visit and the baseline weight. The baseline weights were adjusted as needed to estimate first trimester weight, and the difference between the actual visit dates was used as the denominator as defined by LIFE-Moms.^38^,^39^ If baseline weight was measured at or before 13 weeks of GA, no adjustment was made, as weight at first trimester was assumed to approximate prepregnancy weight in both arms. If baseline weight was measured in the second trimester at 14 weeks of GA, we subtracted 0.45 kg, and if measured at 15 weeks of GA, we subtracted 0.91 kg. Baseline weight was adjusted based on data which showed that women gained on average 1 lb between 10 and 11 weeks and 14 weeks, and 2 lbs between 10 and 11 weeks and 15 weeks.^24^ The weight corresponding to the 36-week assessment was imputed from the last weight obtained before this visit for 11 women (10 preterm deliveries and 1 missing visit).^39^ An additional analysis was conducted only including complete cases.

The IOM guidelines for appropriate GWG specifically refer to second and third trimester GWG. Hence, we computed the outcome as above, but with dates for all baseline weights standardized to 13 weeks, 6 days GA, since little if any weight is gained in the first trimester, and later weeks were already adjusted to reflect first trimester weights. A secondary predefined outcome measure was based on average weekly GWG between the last predelivery weight (measured by the study staff at a study visit, or by UH staff at a prenatal care visit using similar procedures), and enrollment weight. An additional outcome evaluated total GWG between baseline and delivery as above, classified as appropriate or inappropriate.^5^

### Neonatal outcomes

We evaluated gender-adjusted WFL *z*-score using Anthro 3.0.1 WHO software.^40^ To assess growth in premature newborns, Fenton 2013 curves were used to adjust for GA.^41^ In addition, to obtain a weighted growth measure that considers both weight and length, regardless of the GA at birth, we used the Ponderal Index (birthweight in g/length in cm^3^). We classified small for GA and large for GA as birthweight <10th percentile or >90th percentile of the standard, respectively, according to the WHO infant weight charts by gender in full-term newborns. GA adjustment was only performed for premature infants, who were evaluated using gender-adjusted Fenton growth curves. Sex-stratified analyses were preplanned to provide an indication of differences in the effect of the intervention on the main infant outcomes.

### Sample size

Assuming inappropriate GWG of 76% among controls, and that intervention results in 25% reduction of inappropriate GWG, a two-sided alpha of 0.05, and 5% attrition, we estimated that 200 participants would be required to achieve 80% power. PEARLS was designed to enroll participants before 20 weeks of pregnancy, as our site is a referral hospital where women generally present later. The LIFE-Moms Steering Committee subsequently harmonized procedures across the LIFE-Moms study sites, and we were thus required to complete enrollment before 16 weeks of GA. Despite expanding recruitment, over 68% of women who might otherwise have been eligible for PEARLS were excluded due to late presentation, slowing recruitment rates. PEARLS recruitment was stopped by NIH 15 months into the trial, based on recommendations of the LIFE-Moms Data Safety Monitoring Board due to their projected unlikelihood of accruing the target sample size of 200 within the period allowed (32 months).

### Statistical analyses

Descriptive statistics were generated for baseline variables by treatment group. The intent-to-treat principle was followed. Poisson regression evaluated the association between group assignment and binary GWG outcomes; continuous outcomes were evaluated using linear regression, adjusted analysis considered the following variables: Model 1 includes maternal age at baseline; Model 2 includes baseline maternal BMI status; and Model 3 includes maternal age and BMI at baseline. Missingness for GWG was determined to be unrelated to group assignment, and except for one missing 36-week visit, was related to premature delivery. When there was missing data, the last observation carried forward was used for the primary outcome analysis. Analyses were conducted using the Stata Software release 14.

Total PA is reported as the mean low frequency filtered vector magnitude averaged over total awake time (VMU/min) from the wrist-worn accelerometry data.^42^ Mean VMU/min is a reflection of the total movement during awake time. Total PA has been shown to be associated with gestational early insulin response and early infancy fat-free mass.^43^,^44^ Total PA differences at baseline and between 26 and 30 weeks GA were computed. Published software to record and convert the FFQ responses into food and nutrient intake was used.^36^ Changes in mean energy and macronutrient intake are described by treatment groups. Maternal and neonatal adverse events, as per the LIFE-Moms DSMB, are presented by treatment groups.

## Results

**Figure 1:**
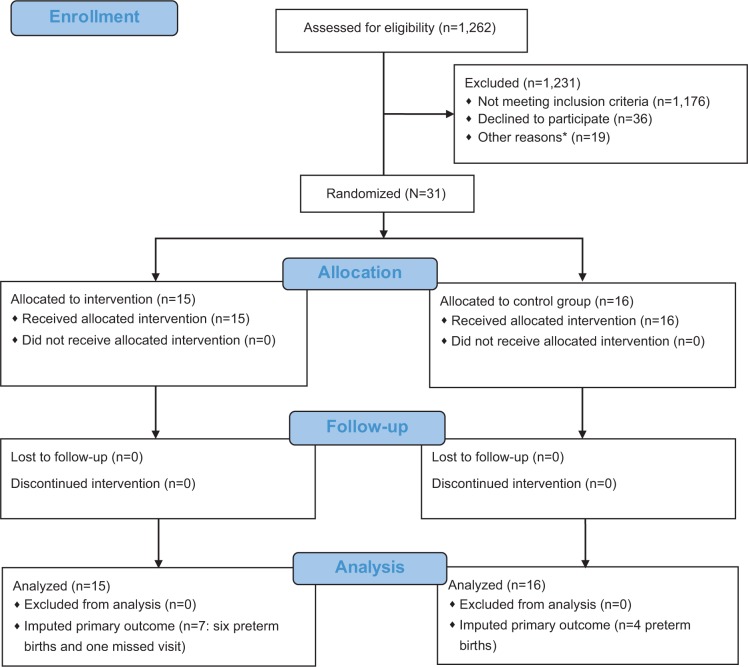
PEARLS recruitment, exclusions and status of enrolled participants. **Notes:** *Other reasons for exclusion: 18 potential eligible women pending at the time of enrollment discontinuation and 1 lost contact during the prescreening phase. **Abbreviation:** PEARLS, Pregnancy and EARly Lifestyle improvement Study.

From January 2013 through March 2014, we screened 1,262 women; 1,176 (93%) did not meet inclusion criteria (Figure 1). The major reasons for ineligibility were as follows: GA ≥16 weeks (68%); BMI <25 kg/m^2^ (12%), or age <18 years (7%) (Table 1). Study retention at delivery was 100%. Thirty-one mothers and their offspring (N=30 excluding 1 missing value) were included in the analyses, 15 in the lifestyle and 16 in the control arm.

Table 2 summarizes sociodemographic, psychosocial, and clinical characteristics by assignment. Most women presented with obesity (22/31, 71%) and were multiparous (22/31, 71%). Median enrollment GA was 14 weeks, 4 days. Mean age at randomization was 27.7 (SD ±5.5) years, 52% (16/31) attended college, 81% (25/31) declared a family income below $20,000/year, 77% (24/31) benefited from supplementary government food programs, 71% (22/31) from WIC, 77% (24/31) were married or had a partner, none reported smoking, and only one reported alcohol consumption while pregnant. Participants in the intervention group were older compared with control group (30.0±5.3 vs 25.6±4.8 years), and the intervention group had more mothers with overweight compared with the controls (7/15, 47% vs 2/16, 13%). A markedly greater proportion in the intervention group identified themselves as Black/African American (7/15, 47% vs 1/16, 6%). However, the effects of race as a social determinant of health among Puerto Ricans is not well characterized, as the ancestral background of the population is a combination of European, West African, and Native American.^45^ Pre-existing hypertension or GDM did not differ between groups. Depressive symptoms were evaluated using the Beck Depression Inventory II (BDI-II) Scale.^34^ Minimal depression was defined as a BDI-II score between 1 and 13 (maximum possible score is 62). All participants reported depressive symptoms: minimal (24/31, 77%) or mild–moderate (7/31, 23%). PA and caloric intake were similar at baseline across allocation groups.

Table 3 summarizes results for primary and secondary outcomes. The confidence limits generally crossed the null, and the results are largely indicative of possible, rather than robust evidence, of underlying effects.

### Predefined primary outcome (baseline to 36 weeks), GWG outcome reflecting second and third trimesters

In the unadjusted model, 27% (4/15) of women in the lifestyle intervention group achieved appropriate GWG weekly target per IOM guidelines compared with 19% (3/16) in the control group (incidence rate ratio [IRR] =1.42; 95% CI: 0.32, 6.35) using intent-to-treat analysis. Similar analysis using only complete case data shows stronger effects. In the intervention group, 67% (10/15) had excess and 7% (1/15) inadequate GWG, compared with 69% (11/16) excess and 13% (2/16) inadequate in the control group (data not shown). The unadjusted association comparing excessive GWG with normal/inadequate GWG is null (IRR =0.97; 95% CI: 0.41, 2.28).

### Appropriate GWG (baseline to delivery)

In the unadjusted model, women assigned to the intervention group appeared twice as likely to achieve a weekly GWG up to delivery within their IOM target (IRR =2.13; 95% CI: 0.39, 11.65) compared with the control group. Since GWG is higher in later weeks, we repeated this analysis excluding the 10 premature deliveries (<37 weeks), and the association became stronger (IRR= 2.67; 95% CI: 0.49, 14.56). Women assigned to the intervention group were more likely to achieve a total GWG up to delivery within their IOM target (unadjusted IRR =1.60; 95% CI: 0.45, 5.67) compared with those in the control group; age and BMI adjusted results were similar (IRR =1.67; 95% CI: 0.40, 6.94). Forty percent (6/15) of women in the intervention achieved appropriate GWG/week compared with 25% (4/16) in the control group. Also, in the intervention group, 27% (4/15) had excess and 33% (5/15) had inadequate GWG, compared with 56% (9/16) excess and 19% (3/16) inadequate in the control group (data not shown).

### Maternal adiposity measurements

The intervention group showed a reduction in thigh circumference between baseline and 36 weeks, whereas the control group showed a slight increase (difference of −5.21 cm between groups; 95% CI: −8.84,−1.59, after adjusting for age and BMI). We did not find differences in the other circumferences.

### Neonatal outcomes

We evaluated 30 live-born infants, of whom 5 in the control and 9 in the intervention group were female. In PEARLS, 91% of the neonatal measures and anthropometric data from clinical records (n=2) were collected within the first 72 hours after birth. The mean birthweight (SD) was 2,890 g (668 g) in the control group and 2,676 g (912 g) in the intervention group. Small for GA was greater in the control group, with four in the control vs one in the intervention group. Only one newborn (intervention group) fulfilled the large for GA criteria.

We did not find a difference in WFL *z*-scores at birth between treatment groups. We observed lower unadjusted WFL *z*-scores in the intervention group compared with the control group only among term male newborns (*z*-score difference: −2.06; 95% CI: −3.41, −0.70). This was attenuated in models adjusted for maternal age and BMI (*z*-score difference: −1.74; 95% CI: −3.04, −0.43). WFL *z*-scores in females were within adequate range for both allocation groups in all models. The difference in Ponderal Index between intervention and control groups adjusting for baseline age and maternal BMI among males was −0.54 (95% CI: −0.91, −0.18), but close to null (0.15) among females (95% CI: −0.45, 0.74). We also performed gender-stratified analysis of the birthweight *z*-score to evaluate if the growth restriction identified in males was associated with insufficient weight gain in the entire cohort including premature newborns (Table 3). We observed differences in growth patterns by gender in weight, but lower birthweight *z*-scores were observed among males in the intervention group. Triceps and thigh skinfolds were slightly lower among males in the intervention group than the controls, while associations were in the opposite direction among females in all skinfolds measures (data not shown). No congenital anomalies were present.

### Diet and PA measures

The intervention group had higher total caloric intake at baseline than the control group, but lower at 36 weeks with 250 calories more reduction between baseline and 36 weeks in the intervention group compared with the control group. The intervention group participants had higher fiber and lower total fat and saturated fat intake than controls at 36 weeks (Table 4). No other differences were noted. The number of observations at 36 weeks was small due to many premature deliveries (32%). Total PA did not differ between groups at either time point but declined from baseline to 26–30 weeks of gestation in both groups.

### Safety-related measures

GDM, eclampsia, and primary C-sections were similar among allocation groups. Preeclampsia (5/15 vs 2/16), prematurity (6/15 vs 4/16), and respiratory morbidity (3/15 vs 1/16) were higher among intervention than control group (Table 5). We did not observe differences in neonatal hypoglycemia. The DSMB consider these events unrelated to the intervention.

## Discussion

PEARLS, a lifestyle intervention framed within a health empowerment model,^22^ combined individual and group sessions with individualized goal setting and feedback, to optimize health behavior and GWG.^29^ Our data suggest that participants receiving the lifestyle intervention were more likely to achieve appropriate GWG within IOM guidelines than the control group. As may be expected, thigh circumference increased in controls,^46^ but decreased in the intervention group; the impact of this finding on mother and infant is unclear. Nevertheless, a recent US longitudinal study assessing the link between maternal adiposity distribution and birthweight concluded that upper-body adiposity was a markedly larger determinant of infant birthweight than lower-body adiposity.^47^ Also, the intervention resulted in greater reduction in caloric intake and some improvement in diet quality. These findings, while not conclusive owing to the small sample size, may facilitate the design of future lifestyle trials. Such studies are of high importance, given the heavy burden of cardiometabolic disease in this population and the role that inappropriate GWG plays in its etiology.

The effects of lifestyle interventions in high-risk pregnancies are unclear. The LIMIT trial, a comprehensive pre- overweight/obesity, did not show any effect on GWG among mainly European-ancestry women.^48^ In ROLO, an Irish randomized control trial to prevent macrosomia in euglycemic secundigravid mothers whose first born child was macrosomic (defined as birthweight >4,000 g), a low glycemic diet initiated in early pregnancy significantly reduced excessive GWG, but not birthweight, their primary outcome.^49^ Wang et al conducted a randomized control trial among 300 pregnant women with overweight, testing the efficacy of regular exercise to prevent GDM onset.^50^ Starting early in pregnancy, women in the intervention group were guided to exercise cycling for at least 30 minutes., three times/week, and achieved a significant reduction in GDM incidence compared with controls (22.0% vs 40.6%; *P*<0.001) and less total GWG (8.38±3.65 vs 10.47±3.33 kg; *P*<0.001). Lower infant birthweight among the intervention group was also seen compared with those in the control group (3,345±397.07 vs 3,457.46±446.00 g; *P*=0.049) in the same study. Another lifestyle intervention study targeting low-income predominantly Latino population in the USA, similarly included diet and PA advice and was framed in a community-based cognitive behavioral theory. This intervention prevented excessive GWG among those within the normal BMI category (40% risk reduction), but not among those with a BMI ≥25 kg/m^2^.^51^

In light of the largely null results of interventions aiming to achieve appropriate GWG among overweight/obese women, maintaining a healthy weight or accomplishing at least a 10% prepregnancy weight reduction has been proposed to reduce obstetrical complications.^52^,^53^ As most pregnancies are unplanned (>60%), prevention programs should ideally target women of reproductive age, which may be difficult, especially within high-risk populations.

Our findings suggest an impact of the intervention on reducing adiposity in male offspring. This was not the result of a generalized reduction in growth, as evidenced by *Z*-scores within an adequate range for the gender-adjusted length growth curves for all infants (data not shown). It is established that the effects of maternal BMI, parity, and GWG on fetal growth vary by gender.^54^ We found a substantial attenuation in the WFL *Z*-scores effect estimate among male newborns, when continuous GWG was added, suggesting that the effect of the intervention on WFL may be partly explained by GWG. In spite of the small sample size, significant gender differences in our WFL outcome highlight the need for further study. Although birthweight is a more intuitive measure, WFL, a standard way of assessing infant overweight/obesity, showed stronger associations at birth compared with birthweight in our study.

Another important observation is that the mean adjusted WFL *Z*-score among male term newborns in controls was within the normal range, while that of the intervention group was below the malnutrition cutoff,^23^,^54^ with the range inclusive of severe malnutrition. However, there was no association between WFL-Z scores and inappropriate GWG nor between WFL-Z scores and obstetrical complications. This is consistent with Eriksson’s findings^55^ indicating a higher susceptibility to intrauterine environment alterations among male newborns toward growth restriction, which may increase future cardiovascular risk. However, our study has limited power.

Access to first-trimester obstetric care in PR is similar to the rest of the USA.^56^ However, women in our study are socially vulnerable, not only because of their race/ethnicity^12^ but also because of their low education level (48%) and low income (81%), confirmed by their supplemental food programs participation (77%). In addition, there was a high prematurity rate in PEARLS (33%), which is aligned with documented disparities in obstetric outcomes among Puerto Ricans, and also explained by the fact that the UH is the main public referral institution for high-risk pregnancies.^12^,^57^

Multiple efforts were made to expand recruitment using diverse strategies including recruitment through WIC offices and advertising through printed, audiovisual and social media, among others. However, the high default GA at the time of first UH visit compared with the target enrollment GA set by the Consortium, and the unwillingness of eligible women being recruited from other settings (e.g. WIC) to change obstetricians, resulted in slower than anticipated recruitment, subsequently leading to early termination of the trial, and low power for these analyses. Accordingly, our results are not conclusive, but provide a base to guide the design of subsequent intervention studies. Table 6 shows sample size estimations to inform the design of future trials in similar high-risk populations, who may especially benefit from prenatal lifestyle interventions.

## Conclusion

Results from this small randomized controlled trial among 31 high-risk pregnant women suggests that a higher percent of women randomized to the diet and PA lifestyle intervention may have some improvement in diet, higher appropriate GWG within IOM guidelines, and lower thigh circumference compared with standard care. These findings, while not conclusive owing to the low power, seem promising and are important to inform future trials needed to reduce the health disparities of this underserved and heavily burdened ethnic group.

## Ethics approval and consent to participate

PEARLS was approved by the University of Puerto Rico Institutional Review Board and by the LIFE-Moms Data and Safety Monitoring Board, the latter of which is an independent regulatory group of experts, convened by the National Institute of Diabetes and Digestive and Kidney Disease (NIDDK). Women agreeing to partake in study procedures for mother-infant dyads provided written informed consent.

## Data sharing statement

The datasets used and/or analyzed during the current study are available from the corresponding author on reasonable request.

## Figures and Tables

**Table 1:** Study exclusions by eligibility criteria

Consortium exclusion criteria	N	%

Gestational age >15 weeks, 6 days^a^	802	68.2
BMI (kg/m^2^) <25	138	11.7
Age <18 years	85	7.2
HbA1c (%) ≥6.5	61	5.2
Known fetal anomaly	0	0
Intention to deliver outside the LIFE-Moms	28	2.4
Consortium hospital Contraindication to aerobic exercise	19	1.5
History of ≥3 consecutive first trimester miscarriages	10	0.9
Unwilling to commit to a 1-year follow-up	8	0.7
Current use of exclusionary medications	7	0.6
Nonviable pregnancy	7	0.6
Multiple pregnancy	4	0.3
Prior or planned bariatric surgery	2	0.2
History of anorexia/bulimia	1	0.1
Current eating disorder	1	0.1
Actively suicidal^b^	1	0.1
Participation in another interventional study	1	0.1
**Site-specific exclusion criterion**		
Past or current intravenous drug user	1	0.1
Self-reported HIV infection^c^	0	0
Inability to fulfill study requirements^d^	0	0
Not Spanish speaking	0	0
Plan on giving up infant for adoption	0	0
**Total exclusions**	1,176	100

**Notes:**

a Calculated from last menstrual period date reported and earliest ultrasound data;

b Assessed by the Beck Depression Inventory II;

c Confirmed from medical records or baseline test;

d Inability to functionally participate in group sessions and other study requirements on a regular basis.

**Abbreviations:** BMI, body mass index; LIFE-Moms, Lifestyle Interventions for Expectant Moms.

**Table 2:** Baseline characteristics

	Overall	Control	Intervention
	N=31	N=16	N=15
	Mean ± SD / n (%)		
**Sociodemographic characteristics**			
Maternal age, years	27.7±5.5	25.6±4.8	30.0±5.3
Educational level			
High school education/diploma or less	15 (48.4)	8 (50.0)	7 (46.7)
College education	16 (51.6)	8 (50.0)	8 (53.3)
Total annual family income			
≤$9,999	14 (45.2)	8 (50.0)	6 (40.0)
$10,000–$19,999	11 (35.4)	5 (31.2)	6 (40.0)
≥$20,000	6 (19.4)	3 (18.8)	3 (20.0)
Any supplementary food program	24 (77.4)	13 (81.3)	11 (73.3)
WIC^a^	22 (71.0)	11 (68.8)	11 (73.3)
Marital status			
Married or living with a partner	24 (77.4)	14 (87.5)	10 (66.7)
Single/separated/divorced/widowed	7 (22.6)	2 (12.5)	5 (33.3)
Race			
Black/African American	8 (25.8)	1 (6.3)	7 (46.7)
White	7 (22.6)	6 (37.5)	1 (6.6)
Other	16 (51.6)	9 (56.2)	7 (46.7)
Hispanic/Latina ethnicity	31 (100.0)	16 (100.0)	15 (100.0)
Current smoking	0 (0.0)	0 (0.0)	0 (0.0)
Any alcohol intake during pregnancy	1 (3.2)	0 (0.0)	1 (6.7)
**Psychosocial characteristics**			
Level of depressive symptoms (BDI-II)^b^			
Minimal (0–13 points)	24 (77.4)	12 (75.0)	12 (80.0)
Mild–moderate (≥14–28 points)	7 (22.6)	4 (25.0)	3 (20.0)
**Clinical characteristics/history**			
Enrollment weight (kg)	91.4±20.8	92.6±19.7	90.1±22.5
Enrollment BMI (kg/m^2^)	35.3±7.4	36.0±7.0	34.6±8.0
Enrollment BMI status			
Overweight	9 (29.0)	2 (12.5)	7 (46.7)
Obese	22 (71.0)	14 (87.5)	8 (53.3)
Gestational age at enrollment (weeks, days): Median (Minimum/Maximum)^c^	14,4 (10,4/16,0)	14,5 (10,6/15,6)	14,4 (10,4/16,0)
Parity			
Primiparous	9 (29.0)	5 (31.2)	4 (26.7)
Multiparous	22 (71.0)	11 (68.8)	11 (73.3)
Blood pressure at screening (mmHg)			
Systolic	114.0±12.0	115.5±13.2	112.4±10.9
Diastolic	68.8±10.6	67.4±9.3	70.2±12.0
Fasting blood glucose mg/dL	92.5±6.4	90.5±4.5	94.7±7.5
Prior hypertension diagnoses with antihypertensive medication use	6 (19.4)	3 (18.8)	3 (20.0)
Prior gestational diabetes mellitus^c^	2/37 (5.4)	1/13 (7.7)	1/24 (4.2)
**Caloric intake/physical activity**			
Total energy intake (kcal/d), n=28	2,473±876	2,523±945	2,415±824
Physical activity (VMU/min),^d,e^ n=28	7,389±3,283	7,773±3,373	6,946±3,252

**Notes:** Participants with a plausible total energy intake (>1,075 kcal and <4,800 kcal) were included.

a WIC: The Special Supplemental Nutrition Program for Women, Infants, and Children;

b BDI-II: Beck Depression Inventory II.

c Denominator includes all previous pregnancies in the studied sample,

d from wrist-worn ActiGraph GT3X+.

e Vector magnitude averaged over total awake time.

**Abbreviations:** BMI, body mass index; WIC, Women, Infants, and Children.

**Table 3:** GWG (classified as appropriate/inappropriate by IOM guidelines) and maternal and neonatal adiposity measurements

Outcome variables	Control mean *±* SD / n (%)	Intervention, mean *±* SD / n (%)	Unadjusted IRR (95% CI)	Model 1, adjusted IRR (95% CI)	Model 2, adjusted IRR (95% CI)	Model 3, adjusted IRR (95% CI)
**MATERNAL OUTCOMES**						

**Second and third trimesters GWG**						

Appropriate weekly GWG: baseline to 36 weeks (n=31)	3 (18.8)	4 (26.7)	1.42 (0.32, 6.35)	1.03 (0.20, 5.30)	1.51 (0.31, 7.45)	1.14 (0.20, 6.67)
*Appropriate weekly GWG: baseline to 36 weeks (Complete case n=20)*	*3 (25.0)*	*4 (50.0)*	*1.53 (0.45, 8.94)*	*1.59 (0.31, 8.14)*	*2.00 (0.43, 9.31)*	*1.51 (0.26, 8.90)*
Appropriate weekly GWG: baseline to delivery (n=31)	2 (12.5)	4 (26.7)	2.13 (0.39, 11.65)	1.04 (0.17, 6.49)	2.20 (0.37, 13.29)	1.14 (0.15, 8.91)
Appropriate total GWG: baseline to delivery (n=31)	4 (25.0)	6 (40.0)	1.60 (0.45, 5.67)	1.59 (0.40, 6.33)	1.69 (0.44, 6.48)	1.67 (0.40, 6.94)

	**Control Mean *±* SD / n (%)**	**Intervention, Mean *±* SD / n (%)**	**Unadjusted Beta coefficient (95% CI)**	**Model 1, adjusted Beta coefficient (95% CI)**	**Model 2, adjusted Beta coefficient (95% CI)**	**Model 3, adjusted Beta coefficient (95% CI)**

**Maternal circumferences (36 weeks visit minus baseline, cm)**						

Neck	0.64±1.35	0.16±1.18	−0.48 (−1.64, 0.67)	−0.11 (−1.32, 1.11)	−0.53(−1.75, 0.68)	−0.09 (−1.42, 1.24)
Arm	−0.09±2.62	−0.50±2.65	−0.40 (−2.76, 1.95)	−0.13 (−2.75, 2.50)	−0.75 (−3.14, 1.65)	−0.62 (−3.39, 2.15)
Thigh	1.98±4.29	−1.29±2.60	−3.27 (−6.61, 0.06)	***−4.26 (−7.82,−0.71)***	***−3.69 (−7.12, −0.25)***	***−5.21 (−8.84,−1.59)***
Hip	4.14±5.33	7.47±7.81	3.33 (−2.40, 9.07)	3.45 (−2.99, 9.89)	3.93 (−2.02, 9.88)	4.47 (−2.41, 11.34)

**Gestational age at birth^a^**	38,4 (34,1/41,1)	37,5 (26,4/40,0)	–	–	–	–

**NEONATAL OUTCOMES**						

**Birthweight for length (WHO WFL *z-*scores)**						

All	−0.05±1.26	−0.59±1.69	−0.54 (−1.80, 0.72)	−0.63 (−2.07, 0.81)	−0.62 (−1.97, 0.74)	−0.76 (−2.34, 0.83)
Females (n=8)	−0.44±1.72	0.52±1.16	0.96 (−1.01, 2.93)	0.27 (−3.09, 3.64)	0.20 (−1.69, 2.09)	−0.83 (−3.85, 2.19)
Males (n=13)	0.08±1.17	−1.98±1.10	***−2.06 (−3.41,−0.70)***	***−1.93 (−3.17, −0.69)***	***−1.96 (−3.42, −0.50)***	***−1.74 (−3.04, −0.43)***

**Birthweight Z-score**						

All	−0.30±0.80	−0.25±0.91	0.05 (−0.57, 0.66)	0.15 (−0.52, 0.82)	−0.02 (−0.69, 0.65)	−0.08 (−0.64, 0.80)
Females (n=14)	−0.65±0.59	−0.02±1.04	0.62 (−0.37, 1.62)	0.85 (−0.35, 2.05)	0.70 (−0.48, 1.88)	0.90 (−0.46, 2.27)
Males (n=16)	−0.12±0.86	−0.60±0.58	−0.47 (−1.25, 0.31)	−0.40 (−1.21, 0.41)	−0.59 (−1.42, 0.24)	−0.52 (−1.38, 0.34)

**Ponderal index (kg/m3)**						

All	2.6±0.3	2.5±0.5	−0.13 (−0.42, 0.16)	−0.17(−0.48, 0.15)	−0.14 (−0.45, 0.18)	−0.18 (−0.52, 0.16)
Females (n=14)	2.5±0.4	2.7±0.4	0.19 (−0.25, 0.63)	0.09 (−0.43, 0.62)	0.24 (−0.28, 0.76)	0.15 (−0.45, 0.74)
Males (n=15)	2.7±0.3	2.1±0.4	***−0.54 (−0.88,−0.20)***	***−0.53 (−0.87, −0.19)***	***−0.56 (−0.92, −0.20)***	***−0.54 (−0.91,−0.18)***

**SGA**	4 (26.7)	1 (6.7)	0.27 (0.03, 2.39)	0.27 (0.03, 2.76)	0.26 (0.03, 2.72)	0.26 (0.02, 3.42)

**LGA**	0 (0.0)	1 (6.7)	–	–	–	–

**Neonatal circumferences (cm) (n=27)**						

Head (growth measure)	32.7±2.5	32.8±2.6	0.18 (−1.76, 2.12)	0.16 (−1.93.,2.26)	−0.15 (−2.15, 1.86)	−0.19 (−2.37, 1.98)
Arm	9.0±0.8	8.9±1.5	−0.11 (−1.01, 0.80)	−0.19 (−1.16, 0.79)	−0.20 (−1.15, 0.75)	−0.30 (−1.32, 0.73)
Abdomen	27.4±2.6	27.9±3.7	0.59 (−1.82, 2.99)	0.56 (−2.04, 3.15)	0.45 (−2.09, 3.00)	0.41 (−2.35, 3.16)
Thigh	13.4±1.3	13.4±2.7	0.03 (−1.56, 1.61)	−0.09 (−1.80, 1.61)	−0.30 (−1.91, 1.31)	−0.47 (−2.20, 1.27)

**Skinfolds (mm) (n=27)**						

Triceps	4.4±1.1	4.6±1.2	0.24 (−0.65, 1.12)	0.01 (−0.90. 0.92)	0.20 (−0.74, 1.14)	−0.04 (−1.01, 0.93)
Subscapular	4.4±0.9	4.6±1.2	0.22 (−0.58, 1.01)	−0.08 (−0.86, 0.70)	0.15 (−0.69, 0.99)	−0.17 (−1.00, 0.65)
Suprailiac	4.0±1.0	4.0±1.0	0.03 (−0.70, 0.76)	−0.02 (−0.81, 0.76)	−0.002 (−0.775, 0.771)	−0.06 (−0.90, 0.77)
Thigh	5.5±1.3	6.0±1.9	0.51 (−0.71, 1.73)	0.29 (−1.00, 1.58)	0.37 (−0.91, 1.64)	0.12 (−1.23, 1.47)

**Notes:** Bold italics highlight significant results. LGA is defined as birthweight <10th percentile or >90th percentile for GA, according to the WHO infant weight charts by gender in full-term newborns; GA adjustment was only performed for premature infants, who were evaluated using gender adjusted Fenton growth curves; Model 1 includes maternal age at baseline; Model 2 includes baseline maternal BMI status; Model 3 includes maternal age and BMI at baseline;

a Median (Minimum, Maximum).

**Abbreviations:** BMI, body mass index; GA, gestational age; GWG, gestational weight gain; IOM, Institute of Medicine; LGA, large for GA; IRR, incidence rate ratio; SGA, small for GA; WFL, weight-for-length.

**Table 4:** Description of daily energy and macronutrients intake across allocation groups and time points (N=17^a^)

Energy/macronutrient	Time point	Control (n=11)	Intervention (n=6)

		Mean ± SD/median (min, max)	Mean ± SD/median (min, max)

Total calorie intake (kcal/d)	Baseline	2,445±956	2,644±1,100
	36 weeks	2,026±1,055	1,971±882
	Change	−419±1,067	−673±1,609
		(median: −73, min: −2,729, max: 1,251)	(median: −115, min: −3,579, max: 694)
Protein (g/1,000 kcal)	Baseline	37.2±4.3	33.6±5.1
	36 weeks	40.8±7.5	40.5±12.6
	Change	3.7±8.8	6.9±11.8
		(median: 0.9, min: −5.3, max: 21.1)	(median: 4.8, min: −5.3, max: 27.1)
Fat (g/1,000 kcal)	Baseline	38.3±5.1	36.6±2.4
	36 weeks	35.4±9.7	30.9±7.9
	Change	−2.9±8.6	−5.7±8.0
		(median: −0.7, min: −12.5, max: 10.1)	(median: −6.8, min: −18.00, max: 3.5)
SFA (g/1,000 kcal)	Baseline	13.3±2.5	12.2±1.5
	36 weeks	14.1±5.0	10.2±1.9
	Change	0.8±3.6	−2.00±2.00
		(median: 0.6, min: −4.4, max: 8.0)	(median: −2.2, min: −4.0, max: 0.8)
MFA (g/1,000 kcal)	Baseline	14.0±1.9	13.3±1.2
	36 weeks	12.2±3.3	11.3±3.1
	Change	−1.8±3.0	−2.0±2.8
		(median: −1.4, min: −5.7, max: 2.2)	(median: −2.2, min: −6.6, max: 1.1)
PUFA (g/1,000kcal)	Baseline	8.0±1.2	8.1±2.3
	36 weeks	6.1±1.4	7.1±2.8
	Change	−1.9±2.2	−1.0±3.3
		(median: −2.3, min: −5.4, max: 1.9)	(median: −1.1, min: −6.1, max: 2.9)
Carbohydrates (g/1,000 kcal)	Baseline	126.6±13.7	136.1±4.8
	36 weeks	132.4±22.9	144.0±28.0
	Change	5.8±21.9	8.0±31.5
		(median: 5.6, min: −26.8, max: 39.7)	(median: 5.6, min: −39.5, max: 52.4)
Fiber (g/1,000 kcal)	Baseline	7.7±2.0	8.8±1.9
	36 weeks	7.9±2.3	11.8±4.1
	Change	0.1±2.4	3.0±2.6
		(median: −0.3, min: −3.6, max: 3.8)	(median: 2.56, min: 0.046, max: 7.89)
Physical activity (n=15^b^)		n=7	n=8
Total physical activity^c^ (VMU/min)	Baseline	7,649.79±4,222.41	8,187.64±3,438.90
	26–30 weeks	4,471.10±3,530.27	5,428.70±4,076.79
	Change	3,178.69±4,792.87	2,758.94±4,573.06
		(median: 4,968.45, min: −2,280.57, max:	(median: 3,949.87, min: −5,386.85, max:
		10,058.07)	10,135.58)

**Notes:** 26–30 weeks by ≥3 valid days.

a Only participants who completed FFQs at baseline and 36 weeks and with a plausible energy intake (>1,075 kcal <4,800) were included in this analysis (33% of the participants delivered prematurely, hence 17 follow-up FFQs were available for comparison).

b Participants who wore the accelerometer at baseline and 26–30 weeks by ≥3 valid days.

c Mean VMU per minute from wrist-worn ActiGraph GT3X+.

**Abbreviations:** SFA, saturated fatty acids; MFA, monounsaturated fatty acids; PUFA, polyunsaturated fatty acids.

**Table 5:** Safety-related measures

Incidence	Overall	Control	Intervention

Maternal			
Gestational diabetes mellitus^a^	6 (19.4)	3 (18.8)	3 (20.0)
Preeclampsia	7 (22.6)	2 (12.5)	5 (33.3)
Eclampsia	2 (6.5)	1 (6.3)	1 (6.7)
Primary C-section	14 (45.2)	7 (43.8)	7 (46.7)
Neonatal			
NICU admission^b,c^	5 (16.1)	2 (12.5)	3 (20.0)
Prematurity (<37 weeks GA)	10 (32.3)	4 (25.0)	6 (40.0)
Neonatal hypoglycemia^d^	4 (13.3)	2 (13.3)	2 (13.3)
Other complications			
Birth trauma^e^	1 (3.3)	0 (0.0)	1 (6.7)
Respiratory morbidity^f^	4 (13.3)	1 (6.7)	3 (20.0)

**Notes:** Medical diagnoses and adverse events abstracted from clinical records.

a Screened between 24 and 27 weeks, diagnose based on the International Association of Diabetes and Pregnancy Study Groups (IADPSG) criteria.

b Denominator = number of pregnancies (N=31).

c NICU: Admission to and stay in the Neonatal Intensive Care Unit or intermediate nursery (Level II) stay of at least 12 hours.

d Newborn with low sugar levels that require intravenous glucose therapy.

e One of several events occurring during birth, which results in damage to tissues or organs (including clavicular fracture, humerus fracture, skull fracture, brachial plexus injury, facial nerve injury, cephalohematoma and other qualifying traumas). One case of cephalohematoma reported.

f Received treatment with supplemental O_2_ and CPAP or ventilator during the first 72 hours of life.

**Abbreviations:** GA, gestational age; CPAP, continuous positive airway pressure.

**Table 6:** Sample size calculations (to achieve a power of 80% for the primary outcome, assuming a two-sided alpha of 0.05 and different proportions projected with adequate GWG)

Outcome	Proportion in control^a^	Proportion in experimental^b^	N in each group

Weekly weight from baseline to 36 weeks	0.19	0.25	748
	0.19	0.27^a^	434
	0.19	0.30	239
	0.19	0.35	120
	0.19	0.40	73
Weekly weight from baseline to delivery	0.13	0.27^a^	127
Total weight from baseline until delivery	0.25	0.40^a^	152

**Notes:**

a Proportion with adequate GWG (IOM guidelines) in PEARLS sample.

b Projected with adequate GWG in PEARLS intervention group.

**Abbreviations:** GWG, gestational weight gain; IOM, Institute of Medicine; PEARLS, Pregnancy and EARly Lifestyle improvement Study.
