# PEARLS Randomized Lifestyle Trial in Pregnant Hispanic Women with Overweight/Obesity: Gestational Weight Gain and Offspring Birthweight [Corrigendum]

**DOI:** 10.2147/DMSO.S247484

**Published:** 2020-02-25

Trak-Fellermeier MA, Campos M, Meléndez M, et al. *Diabetes Metab Syndr Obes*. 2019;12:225–238.

On page 225, Abstract, Results subheading, “Although not statistically significant, women in the intervention group (6/15) were 1.7 times more likely to achieve appropriate weekly GWG until delivery when compared with controls (4/16 women) (adjusted incidence rate ratio = 1.67; 95% CI: 0.40, 6.94).” should read “Although not statistically significant, women in the intervention group (6/15) were 1.7 times more likely to achieve appropriate total GWG until delivery when compared with controls (4/16 women) (adjusted incidence rate ratio = 1.67; 95% CI: 0.40, 6.94).”

Appropriate GWG (baseline to delivery) section, 2^nd^ to 4^th^ line on page 230, “Forty percent (6/15) of women in the intervention achieved appropriate GWG/week compared with 25% (4/16) in the control group.” should read “Forty percent (6/15) of women in the intervention achieved appropriate total GWG compared with 25% (4/16) in the control group.”

The authors apologize for not noticing the inconsistencies between the text and Table 3 earlier.

